# TGF-β1-induced chondrogenesis of bone marrow mesenchymal stem cells is promoted by low-intensity pulsed ultrasound through the integrin-mTOR signaling pathway

**DOI:** 10.1186/s13287-017-0733-9

**Published:** 2017-12-13

**Authors:** Peng Xia, Xiaoju Wang, Yanping Qu, Qiang Lin, Kai Cheng, Mingxia Gao, Shasha Ren, Tingting Zhang, Xueping Li

**Affiliations:** Department of Rehabilitation Medicine, Nanjing First Hospital, Nanjing Medical University, Nanjing, 210006 China

**Keywords:** Low-intensity pulsed ultrasound (LIPUS), Bone marrow mesenchymal stem cells (BMSCs), Differentiation, Integrin, mTOR

## Abstract

**Background:**

Low-intensity pulsed ultrasound (LIPUS) is a mechanical stimulus that plays a key role in regulating the differentiation of bone marrow mesenchymal stem cells (BMSCs). However, the way in which it affects the chondrogenic differentiation of BMSCs remains unknown. In this study, we aimed to investigate whether LIPUS is able to influence TGF-β1-induced chondrogenesis of BMSCs through the integrin-mechanistic target of the Rapamycin (mTOR) signaling pathway.

**Methods:**

BMSCs were isolated from rat bone marrow and cultured in either standard or TGF-β1-treated culture medium. BMSCs were then subjected to LIPUS at a frequency of 3 MHz and a duty cycle of 20%, and integrin and mTOR inhibitors added in order to analyze their influence on cell differentiation. BMSCs were phenotypically analyzed by flow cytometry and the degree of chondrogenesis evaluated through toluidine blue staining, immunofluorescence, and immunocytochemistry. Furthermore, expression of COL2, aggrecan, SOX9, and COL1 was assessed by qRT-PCR and western blot analysis.

**Results:**

We found that LIPUS promoted TGF-β1-induced chondrogenesis of BMSCs, represented by increased expression of COL2, aggrecan and SOX9 genes, and decreased expression of COL1. Notably, these effects were prevented following addition of integrin and mTOR inhibitors.

**Conclusions:**

Taken together, these results indicate that mechanical stimulation combined with LIPUS promotes TGF-β1-induced chondrogenesis of BMSCs through the integrin-mTOR signaling pathway.

## Background

Articular cartilage is composed of extracellular matrix (ECM) and a small number of chondrocytes. The ECM of cartilage comprises mainly aggrecan and collagen, mostly type-I and type-II (COL1 and COL2, respectively). Aggrecan is primarily responsible for maintaining the elasticity and viscosity of transparent cartilage. COL2 accounts for 90% of the composition of the collagen and is generally used as a marker of chondrogenesis, whereas COL1 is used to identify osteogenesis [1]. Excessive protease hydrolysis of cartilage ECM is the key step involved in the destructive process caused by sports injuries, accidental trauma, or aging, and regularly progresses to more serious joint disorders including osteoarthritis (OA) [1]. Following traumatic or pathological injury, articular cartilage, the load-bearing tissue of joints, has a very limited capacity for repair. Even minor lesions or injuries may trigger progressive damage and joint degeneration [2].

Tissue engineering-based cartilage repair is among best of the current treatments for articular cartilage damage. Autologous chondrocyte implantation (ACI) and matrix-induced autologous chondrocyte implantation (MACI) offer potential regeneration of cartilage over the long term. However, due to the limitations and disadvantages of ACI, alternative therapies for cartilage regeneration are desirable [3]. The availability of large quantities of bone marrow mesenchymal stem cells (BMSCs) which can undergo multilineage differentiation, notably chondrogenic differentiation, has made them the most promising cell source for cartilage regeneration [4].

BMSC cultures can easily be prepared from patients without invasive surgery. They are isolated through bone marrow harvest using a trephine needle from the hip or sternum. These cells grow rapidly, retaining their capacity to differentiate into chondrocytes under certain conditions [5]. Several factors such as transforming growth factor beta (TGF-β) and SRY-related high mobility group-box gene 9 (SOX9) are involved in chondrogenic differentiation, proliferation, and maintenance [6, 7]. TGF-β is a polypeptide growth factor, a macromolecular complex found in bone, cartilage matrix, and platelets, which can induce differentiation of primitive mesenchymal stem cells to form cartilage tissue in embryos [8]. SOX9 is a factor required for the formation of cartilage, the lack of which leads to achondroplasia [9]. Some studies have found that SOX9 directly activates the expression of COL2, COL1, and aggrecan in mesenchymal stem cells [10, 11].

Mechanical stress effectively regulates BMSC differentiation. Different forms of mechanical stress, including shear stress, cellular stretch and centrifugal force, are known to affect BMSC differentiation in various ways [12–14]. Low-intensity pulsed ultrasound (LIPUS) provides mechanical stress and can be used to promote cartilage repair [15]. It has been demonstrated that treatment of mature chondrocytes with LIPUS enhances their expression of matrix genes, such as COL2 and aggrecan [16]. Some studies have suggested that LIPUS influences the differentiation of BMSCs [17, 18] and an in vitro study showed that LIPUS can facilitate TGF-β-mediated chondrogenic differentiation of BMSCs [19].

Integrins are a type of transmembrane cell surface stress receptor that interact with ECM and play an important role in mediating intracellular signaling transduction [20]. Activation of β1 integrin, expressed principally on the membranes of chondrocytes, accelerates their differentiation and maturation to promote cartilage formation and remodeling via regulation of ECM synthesis [21, 22]. In addition, the biological function of BMSCs can be activated through mechanical stress stimulation of integrins on the cell membrane surface [23]. Our previous study demonstrated that LIPUS acts as mechanical stress that can increase the expression of COL2 and aggrecan via the integrin signaling pathway, leading to the proliferation of chondrocytes [24].

The mechanistic target of Rapamycin (mTOR) signaling pathway is located downstream of the PI3K/Akt pathway and plays a key role in regulating cell proliferation, apoptosis, and transformation [25]. Although some studies have demonstrated that mTOR is important in regulating the differentiation of BMSCs [26], little is known about whether the effects of LIPUS on their chondrogenic differentiation could be mediated by the integrin-mTOR signaling pathway.

In this study, we aimed to investigate whether LIPUS affects TGF-β-mediated chondrogenic differentiation of BMSCs through the integrin-mediated mTOR signaling pathway. We hypothesize that LIPUS can promote the chondrogenesis of BMSCs which have been treated with TGF-β through the integrin-mTOR signaling pathway.

## Methods

The experimental protocol relating to rats was in accordance with the US National Institutes of Health’s Guidelines of Laboratory Animal Use and approved by the Nanjing Medical University Ethics Committee of Nanjing Hospital (20150829).

### Reagents

Phosphate-buffered saline (PBS), 0.25% trypsin, 0.2% type-II collagenase, basic medium (containing hexadecadrol 0.1 μmol/L, Vitamin C 0.1 mmol/L, ascorbate 2-phosphate 50 g/mL, 0.35 mM proline, 1 mM pyruvate, ITS Premix 50 mg/mL, insulin 6.25 g/mL, transferrin 6.25 g/mL, sodium selenate 6.25 g/mL, linoleic acid 5.35 g/mL, Dulbecco’s modified Eagle’s medium (DMEM) with 10% fetal bovine serum (FBS), 50 units/mL penicillin and 50 mg/mL streptomycin), immunofluorescence and immunocytochemical kits, toluidine blue, total protein extraction kits, qRT-PCR–related reagents including Qiagen RNeasy Mini Kits and qPT-PCR kits, and other cell isolation and culture medium-related supplies were purchased from KeyGEN (Nanjing, Jiangsu, China). Rabbit anti-rat antibody against CD44 and CD45 were purchased from Santa Cruz Biotechnology (Dallas, TX, USA). Fluorescein isothiocyanate (FITC)-rabbit anti-rat antibodies against CD34 and CD90 were purchased from Abcam (Cambridge, MA, USA) and FITC-goat anti-rabbit IgG was purchased from Jackson (Philadelphia, PA, USA). FITC-conjugated isotype-matched mouse IgG1 was purchased from R&D Systems Inc. (Minneapolis, MN, USA) and rabbit polyclonal IgG was purchased from Epitomics (Burlingame, CA, USA). Rabbit anti-rat antibodies against COL2, COL1, SOX9, aggrecan, integrin β1, phosphorylated-mTOR (p-mTOR), and β-actin were purchased from Acris (Herford, Germany) and goat anti-rabbit (Fab)_2_ secondary antibody was purchased from Santa Cruz Biotechnology (Dallas, TX, USA). Integrin inhibitor glycine-arginine-glycine-aspartic acid-serine-proline (GRGDSP) was purchased from AnaSpec (Fremont, CA, USA) and mTOR inhibitor. Rapamycin was purchased from Selleck Chemicals, (Houston, TX, USA). TGF-β1 was purchased from PeproTech (Rocky Hill, NJ, USA).

### Cell culture

BMSCs were obtained from 8-week-old male Sprague-Dawley (SD) rats [27, 28]. Bone marrow was flushed from the bone cavity of the femurs and tibias of the rats with DMEM containing 10% FBS. The non-solid fraction of the bone marrow was placed into 10-mL centrifuge tubes, then 3 mL PBS was added and centrifuged for 10 minutes (1000 rpm). The supernatant and fat were discarded and the remaining cells were washed with PBS by centrifugation for 10 minutes (1000 rpm), twice. After washing, 5 mL of DMEM were added to the cell pellet, which was resuspended, and the cells then seeded onto petri dishes in 5% CO_2_/95% air at 37 °C. The BMSCs were identified by flow cytometric analysis of phenotype. When cells reached 30% to 40% confluence, dead cells were removed and the medium was replaced with fresh DMEM. When the cells reached 80–90% confluence, they were split and subcultured at a density of approximately 2 × 10^6^ cells per culture dish. The morphology of BMSCs was observed using a light microscope (×200 magnification) [29].

### Flow cytometric analysis of BMSC phenotype

The surface marker expression of BMSCs isolated from rats was analyzed by flow cytometry, including CD90, CD44, CD45, and CD34. CD34 and CD45 are markers of hemopoietic progenitor cells, whereas CD44 and CD90 are markers of mesenchymal cells. Briefly, the BMSCs were harvested by trypsinization, washed twice in PBS, then pelleted by centrifugation at 400 g for 5 minutes at room temperature and re-suspended in the staining buffer at 2 × 10^6^/mL for 15 minutes at 4 °C. One hundred microliters of cell suspension were incubated with FITC-conjugated primary antibodies against rabbit CD90 and CD34, and unconjugated antibodies to CD44 and CD45 for 15 minutes at 4 °C. Unbound antibodies were washed away by adding ice-cold staining buffer. The cell pellet was resuspended in the staining buffer containing FITC-conjugated goat anti-rabbit immunoglobulin G (IgG) for CD44 and CD45 for at least 15 minutes at 4 °C. The cells were then washed with ice-cold PBS containing 2% bovine serum albumin (BSA) before analysis using a fluorescence-activated cell sorter (FACS Calibur, BD Biosciences, San Jose, CA, USA). FITC-conjugated isotype-matched mouse IgG1 was used as the isotype control for both CD90 and CD34. Rabbit polyclonal IgG was used as the isotype control for both CD44 and CD45. The percentage of positive cells was analyzed using WinMDI 2.8 software (The Scripps Institute, West Lafayette, IN, USA).

### Treatment with TGF-β and application of low-intensity pulsed ultrasound

A second generation of BMSCs were cultured with basic medium (containing hexadecadrol 0.1 μmol/L, Vitamin C 0.1 mmol/L, ascorbate 2-phosphate 50 g/mL, 0.35 mM proline, 1 mM pyruvate, ITS Premix 50 mg/mL, insulin 6.25 g/mL, transferrin 6.25 g/mL, sodium selenate 6.25 g/mL, linoleic acid 5.35 g/mL, DMEM with 10% FBS, 50 units/mL penicillin and 50 mg/mL streptomycin) in six-well culture dishes at a density of 1 × 10^4^ cells in 2 mL fluid per well (diameter = 34.8 mm, surface area = 9.5 cm^2^, thickness of the well bottom = 1.2 mm). In order to demonstrate that the effect of TGF-β1 on the chondrogenesis of BMSCs is dose-dependent, varying doses (0.1 ng/mL, 1 ng/mL, 10 ng/mL) of TGF-β1 were added to the wells. Dead cells were removed and the medium replaced with fresh chondrogenic medium (basic medium supplemented with TGF-β1) every 3 days. The most appropriate dosage of TGF-β1 for the chondrogenesis of BMSCs was selected according to the immunofluorescence of COL2, toluidine blue staining, and qRT-PCR analysis of COL2, COL1, SOX9, and aggrecan.

**Fig. 1:**
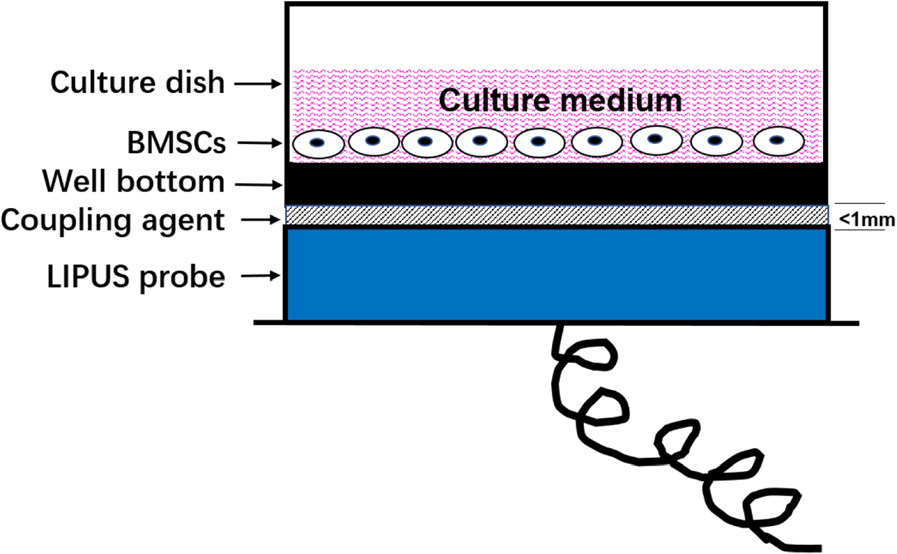
LIPUS stimulation of BMSCs in vitro. A layer of coupling agent (< 1 mm thick) was applied between the LIPUS probe and culture dish, the probe placed below the tissue culture dish. BMSCs were treated once a day for 10 days at a duty cycle of 20%, at 3 MHz for 20 minutes in a 37 °C incubator in a humidified atmosphere with 5% CO_2_ in air. *LIPUS* low-intensity pulsed ultrasound. *BMSCs* bone marrow mesenchymal stem cells, *LIPUS* low-intensity pulsed ultrasound

The LIPUS experiment was performed following selection of the TGF-β1 dosage. The second generation of BMSCs were cultured with chondrogenic medium in six-well culture dishes at a density of 1 × 10^4^ cells in 2 mL fluid per well in 5% CO_2_/95% air at 37 °C. A coating of coupling agent (<1 mm thick) was applied between the LIPUS probe and culture dish, the probe being placed below the culture dish (Fig. 1). The BMSCs were then treated with LIPUS (HT2009-1, Ito Corporation, Tokyo, Japan, unfocused plane average intensities of 20 mW/cm^2^, 30 mW/cm^2^, 40 mW/cm^2^ and 50 mW/cm^2^) for 20 minutes/day in a 37 °C incubator in a humidified atmosphere containing 5% CO_2_ in air for 10 days.

### Application of integrin and mTOR inhibitors

To investigate the role of integrin and mTOR on the effects of LIPUS on BMSCs, the second-generation cells were incubated with the specific integrin inhibitor GRGDSP (1 μM, 5 μM and 10 μM) [30] and the mTOR inhibitor Rapamycin (1 μM, 5 μM and 10 μM) [31], both exposed and not exposed to LIPUS, for 10 days. The BMSCs cultured with chondrogenic medium were divided into six groups: control group, LIPUS group, GRGDSP group, GRGDSP + LIPUS group, Rapamycin group and, Rapamycin + LIPUS group.

### Immunofluorescence

To evaluate the distribution of COL2 protein, cells were seeded into six-well culture dishes at a density of 1 × 10^4^ cells/well then cultured in basic or chondrogenic medium (basic medium dosed with 0.1 ng/mL, 1 ng/mL, or 10 ng/mL TGF-β1) prior to immunofluorescence (IF) staining. After 10 days, both TGF-β-treated and control cells were washed briefly in PBS, three times. Cells were then fixed in cold 4% paraformaldehyde for 15 minutes at room temperature, and blocked in goat serum albumin for 15 minutes. Plates were subsequently incubated overnight at 4 °C with rabbit polyclonal antibody to COL2. Sequentially, plates were incubated with secondary antibodies conjugated to FITC, for 1 hour at room temperature. After rinsing in PBS, cells were viewed and imaged using a Dmi 6000-B fluorescence microscope (Leica, Brunswick, Germany).

### Toluidine blue staining

To determine the presence of glycosaminoglycans (a key marker of chondrogenesis), cells were washed with PBS three times and fixed in 4% paraformaldehyde at room temperature for 20 minutes, followed by three additional washes with PBS. The cells were then stained with toluidine blue for 30 minutes, washed with PBS and observed using an inverted microscope.

### qRT-PCR analysis

We investigated relative concentrations of the mRNA of glyceraldehyde 3-phosphate dehydrogenase (GAPDH), chondrogenic genes including COL2, aggrecan, and SOX9, and the osteogenic gene COL1 in BMSCs using quantitative real-time PCR (qRT-PCR). Total RNA was extracted with Trizol, treated with DNase and column purified using a Qiagen RNeasy Mini Kit. Complementary DNA was synthesized using Superscript III from 1 μg total RNA following the manufacturer's instructions. PCR primers (Table 1) were designed based on cDNA sequences from the NCBI Sequence database using Primer Express® software, and primer specificity confirmed using BLASTN searches. qRT-PCR was performed using an ABI Prism 7500 Fast Real-Time PCR System (Applied Biosystems, Foster City, CA, USA) using SYBR Green as the detection reagent. Briefly, 2 μL of template cDNA, 20 pmol of gene-specific primer and 10 μL of 2 × Master Mix were used in a 20 μL reaction volume. Each sample was tested in duplicate. The thermocycling conditions were as follows: 1 cycle for 15 minutes at 95 °C for activation of polymerase, 40 cycles of 10 s at 95 °C and 1 minute at 60 °C for amplification. Dissociation curve analysis was carried out to verify the absence of primer dimers and/or non-specific PCR products. GAPDH was used as the housekeeping gene. To quantify the relative expression of each gene, Ct values were normalized against the endogenous reference (ΔCt = Ct_target_ – Ct_GAPDH_) and were compared with a calibrator using the 2^-ΔΔCt^ method (ΔΔCt = ΔCt_sample_ –ΔCt_calibrator_).

**Table 1:** Primer sequences for qRT-PCR

Gene	Primer sequences
GAPDH	Forward:5′-ACCACAGTCCATGCCATCAC-3′
	Reverse:5′-TCCACCACCCTGTTGCTGTA-3′
COL2	Forward:5′- CTCAAGTCGCTGAACAACC -3′
	Reverse:5′- CTATGTCCACACCAAATTCC -3′
Aggrecan	Forward:5′-AGGATGGCTTCCACCAGTGC-3′
	Reverse:5′-TGCGTAAAAGACCTCACCCTCC-3′
COL1	Forward:5′- ATGTTCAGCTTTGTGGAC -3′ Reverse:5′- GGATGCCATCTTGTCCAG -3′
SOX9	Forward:5′- GACGTGCAAGCTGGGAAAGT-3′
	Reverse:5′- CGGCAGGTATTGGTCAAACTC-3′

*COL2* type II collagen, *COL1* type I collagen, *GAPDH* glyceraldehyde 3-phosphate dehydrogenase, *SOX9* sex-determining region Y-box 9

### Immunocytochemistry

BMSCs were fixed with 4% paraformaldehyde for 30 minutes, washed with PBS three times, incubated with 3% H_2_O_2_-methanol solution at room temperature for 10 minutes, washed with PBS three times, blocked then incubated with goat serum (50–100 μL) at room temperature for 20 minutes. Cells were then incubated with COL2 antibody (50–100 μL of a 1:200 dilution) at 37 °C for 2 hours and washed with PBS three times before the addition of 50 μL of an intensifier, which was incubated at room temperature for 30 minutes. Cells were subsequently washed with PBS three times, incubated with horseradish peroxidase (HRP)-conjugated anti rabbit-(Fab)_2_ antibody (50 μL) at 37 °C for 30 minutes, washed with PBS three times, followed by color development using DAB and stained with hematoxylin. Three culture dishes were evaluated per condition and three areas with positively-stained cells were selected. COL2 expression was viewed and images acquired using a light microscope.

### Western blot analysis

Cells were collected and the expression of COL2, aggrecan, SOX9, COL1, β1 integrin, p-mTOR, and β-actin determined using western blot analysis. Proteins were extracted from the chondrocytes using a total protein extraction kit of which 20–25 μg/well were loaded onto sodium-dodecyl sulfate polyacrylamide gel for electrophoresis and electroblotted onto nitrocellulose membranes. Membranes were blocked with skimmed milk for 2 hours and incubated at 4 °C overnight with the following primary antibodies: anti-COL2 (1:500 dilution), anti-COL1 (1:500 dilution), anti-aggrecan (1:500 dilution), anti-SOX9 (1:500 dilution), anti-β1 integrin (1:500 dilution), anti-phospho-mTOR (1:1000 dilution), and anti-β-actin (1:500 dilution). Cells were subsequently washed three times with Tween-20 in PBS and incubated with peroxidase-conjugated goat anti-rabbit IgG secondary antibody (1:5000 dilution) at 37 °C for 2 hours, followed by a further three washes as performed above. Membranes were developed following exposure to chemiluminescence reagents (ECL kit).

### Statistical analysis

Three independent experiments were performed for each set of results. All data were expressed as mean ± standard error of the mean (SEM) and analyzed using SPSS 21.0 software (IBM Corp, Armonk, NY, USA). The differences between groups were analyzed by single-factor analysis of variance (ANOVA) and pair-wise Student’s *t* test. A *P* value <0.05 was considered statistically significant.

## Results

### General observation and identification of BMSCs

**Fig. 2:**
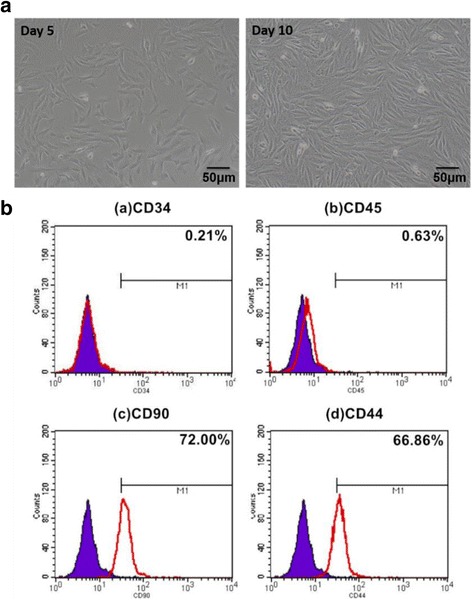
General observation and identification of BMSCs and TGF-β1-induced chondrogenesis. **a** Second generation of BMSCs cultured in basic medium and observation of cells in vitro by microscopy on days 5 and 10; scale bars = 50 μm. **b** Flow cytometry histogram of BMSCs. *Purple shaded area* represents control, *red curve* represents cells positive for CD markers. Graphs a, b, c, d separately indicate markers of CD34 (0.21 ± 0.03%), CD45 (0.63 ± 0.09%), CD90 (72.00 ± 0.67%), and CD44 (66.86 ± 0.36%), respectively

The second generation BMSCs cultured in basic medium on days 5 and 10 are shown in Fig. 2a. The BMSCs appear as fusiform or triangular cells. BMSCs were identified using flow cytometry, a representative histogram of which is shown in Fig. 2b. BMSCs were positive for CD44 (66.86 ± 0.36%), CD90 (72.00 ± 0.67%), and negative for CD34 (0.21 ± 0.03%) and CD45 (0.63 ± 0.09%).

### TGF-β1-induced chondrogenesis of BMSCs

**Fig. 3:**
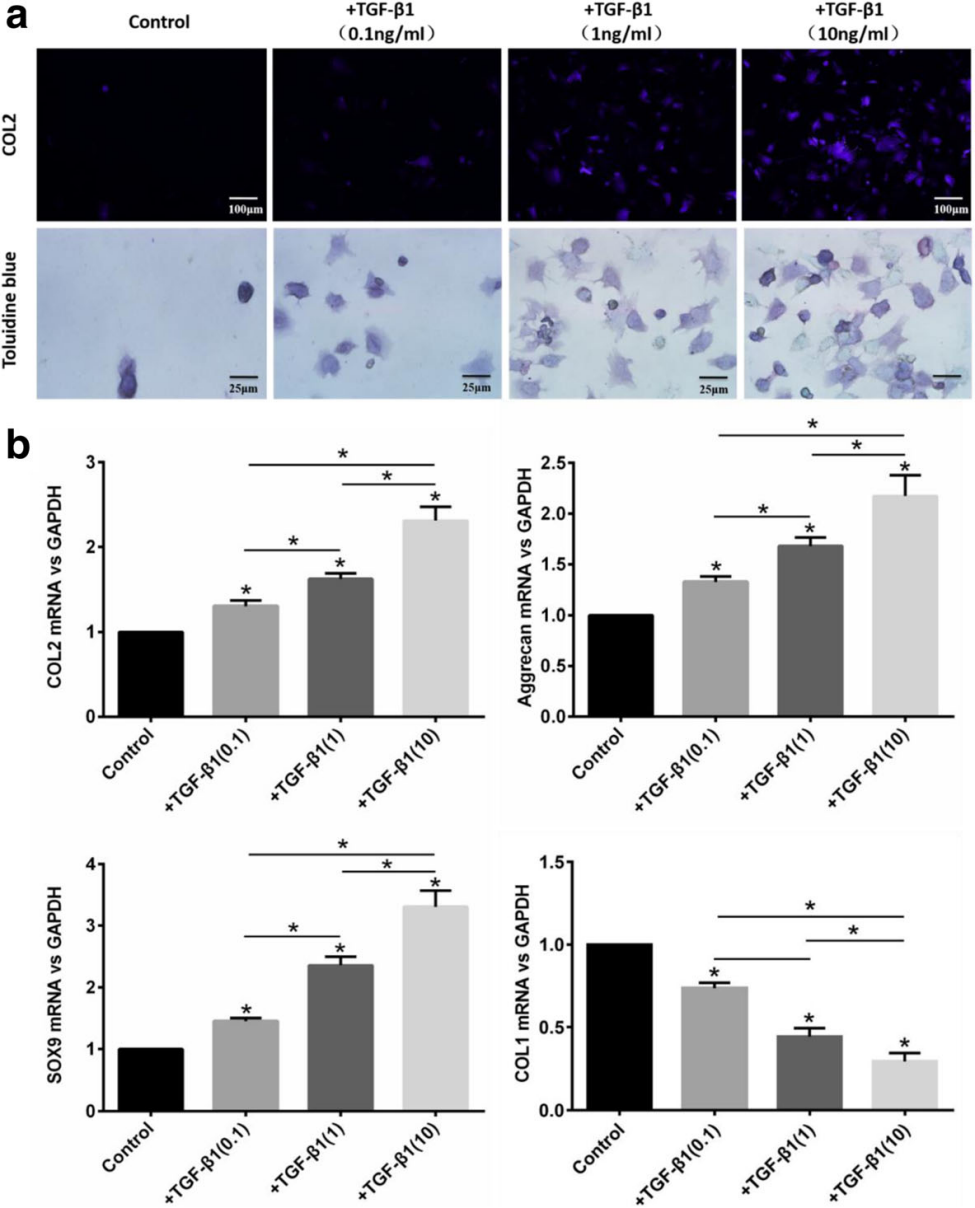
Identification of TGF-β1-induced chondrogenesis. **a** Immunofluorescence staining of COL2 and toluidine blue staining in BMSCs cultured with basic or chondrogenic medium (basic medium treated with different doses of TGF-β1) on day 10; scale bars = 100 μm or 25 μm. **b** qRT-PCR analysis of COL2, aggrecan, SOX9, and COL1. The values are mean ± SEM of triplicate experiments. n = 4; *P < 0.05. *COL1* collagen type-I, *COL2* collagen type-2. *GAPDH* glyceraldehyde 3-phosphate dehydrogenase, *LIPUS* low-intensity pulsed ultrasound, *SOX9* SRY-related high mobility group-box gene 9, *TGF* transforming growth factor

To establish an appropriate dose of TGF-β1 for the induction of chondrogenic differentiation of BMSCs, COL1, and COL2 immunofluorescence staining of BMSCs cultured with medium containing different doses of TGF-β1 was evaluated on day 10, as was toluidine blue staining and expression of COL2, aggrecan, SOX9, and COL1 mRNA. The results of immunofluorescence staining demonstrated that COL2 protein was expressed in BMSCs following TGF-β1 treatment, and COL2 positivity was greater after treatment with 10 ng/mL TGF-β1 than any other group on day 10 (Fig. 3a). The blue cytoplasmic staining of BMSCs in the toluidine blue assay demonstrated the same result (Fig. 3a), as did the mRNA expression of COL2 (*P* = 0.005 (0.1 ng/ml), *P* < 0.001 (1 ng/ml), *P* < 0.001 (10 ng/ml)), aggrecan (*P* = 0.008 (0.1 ng/mL), *P* < 0.001 (1 ng/ml), *P* < 0.001 (10 ng/ml)), and SOX9 (*P* < 0.001 (0.1 ng/mL), *P* < 0.001 (1 ng/ml), *P* < 0.001 (10 ng/ml)), which increased significantly, and COL1 (*P* < 0.001 (0.1 ng/mL), *P* < 0.001 (1 ng/ml), *P* < 0.001 (10 ng/ml)) that was decreased (Fig. 3b).

### LIPUS promoted TGF-β1-induced chondrogenesis of BMSCs

**Fig. 4:**
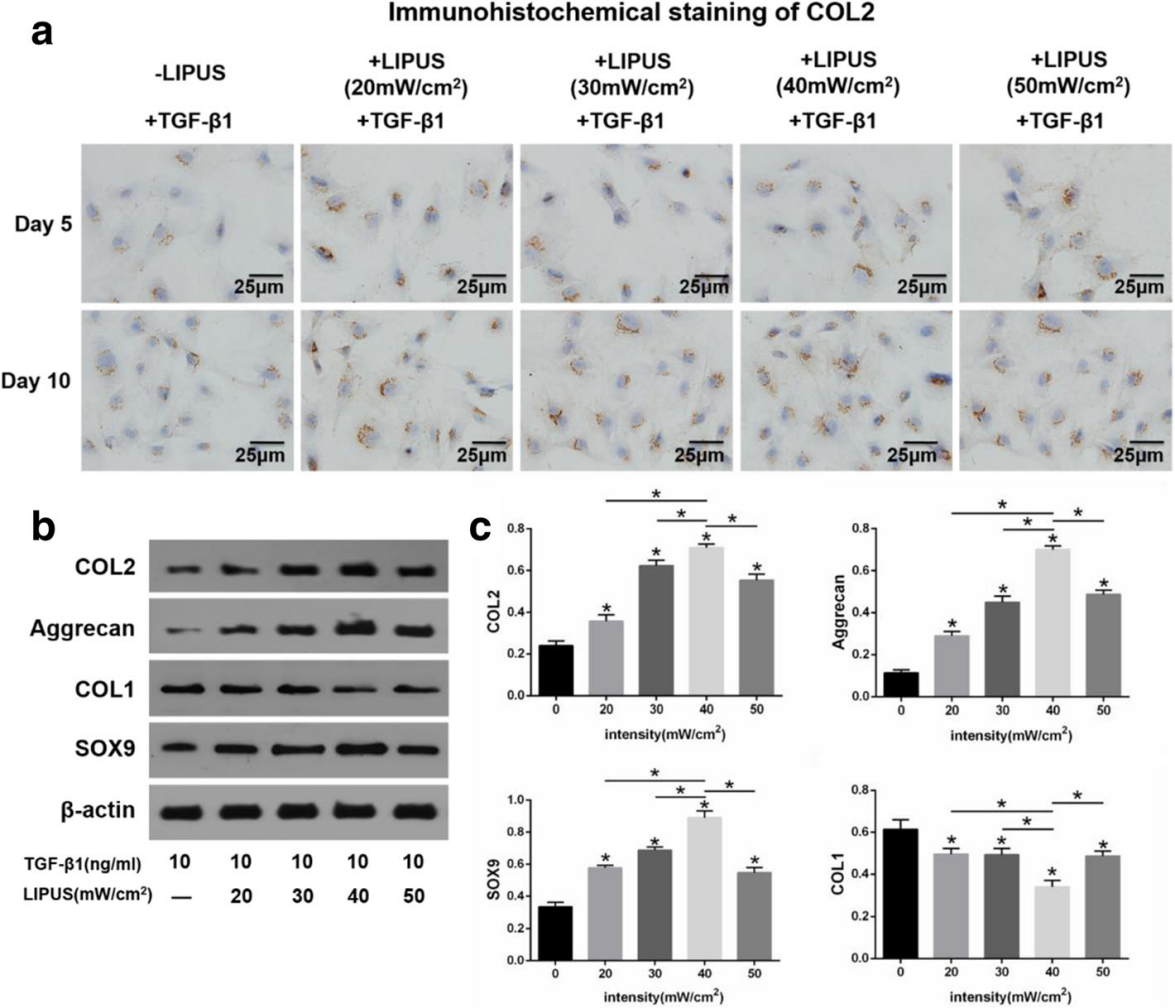
LIPUS promoted TGF-β1-induced chondrogenesis of BMSCs. **a** Immunocytochemistry staining of COL2 in BMSCs cultured with 10 ng/ml TGF-β1-treated medium after LIPUS stimulation at four intensities on days 5 and 10. Images were acquired using a microscope; scale bars = 25 μm. **b** Western blot analysis of COL2, aggrecan, SOX9, and COL1 with β-actin as a loading control. **c** Statistics of the western blot results of COL2, aggrecan, SOX9, and COL1. The ratio between the target and β-actin was used to normalize the data for comparison. Values are the mean ± SEM of triplicate experiments. n = 4; *P < 0.05. *COL1* collagen type-I, *COL2* collagen type-2. *LIPUS* low-intensity pulsed ultrasound, *SOX9* SRY-related high mobility group-box gene 9, *TGF* transforming growth factor

Immunocytochemical staining of COL2 in BMSCs treated with TGF-β1 following exposure to LIPUS of different intensities (20 mW/cm^2^, 30 mW/cm^2^, 40 mW/cm^2^, or 50 mW/cm^2^) for 20 minutes on days 5 and 10 are shown in Fig. 2a. The results demonstrate that there were a greater number of COL2-positive cells following LIPUS compared to the BMSCs that had received no exposure (Fig. 4a).

To determine the effects and the appropriate intensity of LIPUS on the chondrogenic differentiation of BMSCs, we examined COL2, aggrecan, SOX9, and COL1 protein expression after LIPUS stimulation at four different intensities (20 mW/cm^2^, 30 mW/cm^2^, 40 mW/cm^2^, and 50 mW/cm^2^) in BMSCs treated with TGF-β1. Western blot analysis demonstrated that the expression of COL2 (*P* = 0.01 (20 mW/cm^2^), *P* < 0.001 (30 mW/cm^2^), *P* < 0.001 (40 mW/cm^2^), *P* < 0.001 (50 mW/cm^2^)), aggrecan (*P* < 0.001 (20 mW/cm^2^), *P* < 0.001 (30 mW/cm^2^), *P* < 0.001 (40 mW/cm^2^), *P* < 0.001 (50 mW/cm^2^)), and SOX9 (*P* < 0.001 (20 mW/cm^2^), *P* < 0.001 (30 mW/cm^2^), *P* < 0.001 (40 mW/cm^2^), *P* < 0.001 (50 mW/cm^2^)) increased significantly, but the expression of COL1 (*P* = 0.029 (20 mW/cm^2^), *P* = 0.026 (30 mW/cm^2^), *P* < 0.001 (40 mW/cm^2^), *P* = 0.02 (50 mW/cm^2^)) decreased significantly following LIPUS stimulation at all four intensities, especially at 40 mW/cm^2^ (Fig. 4b and c).

### Inhibition of integrin-mTOR pathway suppressed TGF-β1-induced chondrogenesis of BMSCs

**Fig. 5:**
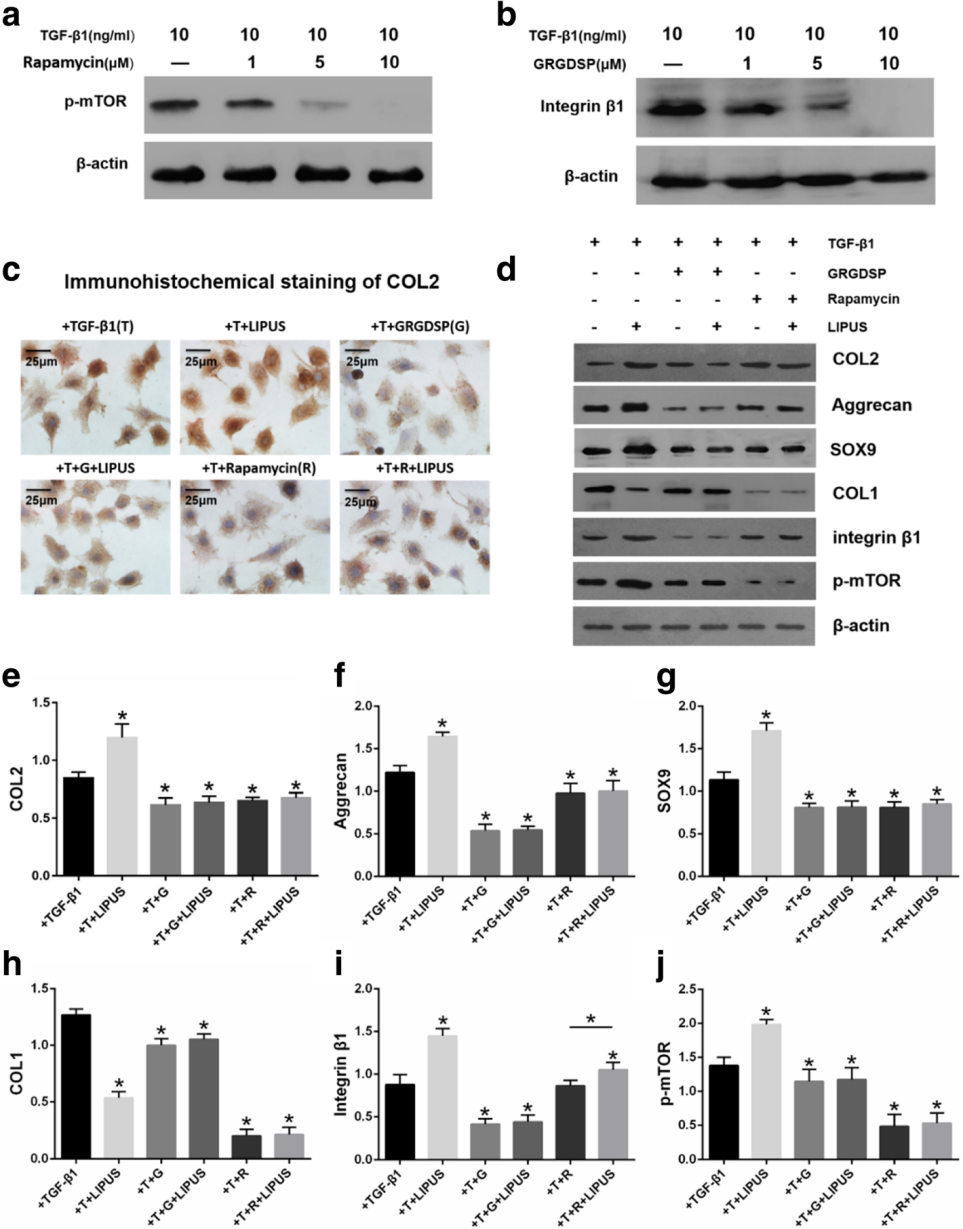
Inhibition of integrin-mTOR pathway suppressed TGF-β1-induced chondrogenesis of BMSCs and the treatment effects of LIPUS. **a** Western blot analysis demonstrate that GRGDSP inhibited β1 integrin expression in a dose-dependent manner. **b** Western blot results show that Rapamycin inhibited the activation of mTOR in a dose-dependent manner. **c** Immunohistochemical staining for COL2 of BMSCs cultured with TGF-β1-treated medium in control, LIPUS, GRGDSP, GRGDSP + LIPUS, Rapamycin, and Rapamycin + LIPUS groups. Images acquired by microscopy on day 10; scale bars = 25 μm. **d** Western blot analysis of COL2, aggrecan, SOX9, COL1, integrin β1, and p-mTOR with β-actin as a loading control in BMSCs treated with TGF-β1 with or without LIPUS stimulation following GRGDSP or Rapamycin treatment. **e**–**j** Statistics of the western blot results of COL2, aggrecan, SOX9, COL1, β1 integrin, and p-mTOR. The ratio between the target and β-actin was used to normalize the data for comparison. The values are mean ± SEM of triplicate experiments. n = 6; *P < 0.05. *COL1* collagen type-I, *COL2* collagen type-2. *LIPUS* low-intensity pulsed ultrasound, *mTOR* mechanistic target of rapamycin, *SOX9* SRY-related high mobility group-box gene 9, *TGF* transforming growth factor

The effects of integrin inhibitor GRGDSP were dose-dependent (Fig. 5a). We examined the expression of COL2, aggrecan, SOX9 and COL1 in BMSCs treated with TGF-β1 after incubation with GRGDSP. The western blot results are shown in Fig. 5d. After GRGDSP treatment, COL2 (*P* = 0.015), aggrecan (*P* < 0.001), SOX9 (*P* = 0.008), and COL1 (*P* = 0.005) expression decreased significantly (Fig. 5e–j).

The effects of the mTOR inhibitor Rapamycin were also dose-dependent (Fig. 5b). Its effects on the expression of COL2, aggrecan, SOX9, and COL1 in BMSCs treated with TGF-β1 are shown in Fig. 4d. After Rapamycin treatment, COL2 (*P* = 0.029), aggrecan (*P* = 0.004), SOX9 (*P* = 0.009), and COL1 (*P* = 0.017) expression decreased significantly (Figs. 5e–j).

### Intergrin-mTOR pathway inhibitor suppressed the effects of LIPUS on TGF-β1-induced chondrogenesis of BMSCs

Immunohistochemical staining of COL2 in BMSCs treated with TGF-β1 in the LIPUS group was much stronger than that in the control, GRGDSP, GRGDSP + LIPUS, Rapamycin, and Rapamycin + LIPUS groups. The staining in GRGDSP, GRGDSP + LIPUS, Rapamycin, and Rapamycin + LIPUS groups was weaker than that in the control group. It was not possible to distinguish any difference in COL2 staining between the GRGDSP, GRGDSP + LIPUS, Rapamycin, and Rapamycin + LIPUS groups (Fig. 5c).

The western blot results of COL2, aggrecan, SOX9, COL1, β1 integrin, and p-mTOR are shown in Fig. 5d. Densimetric analysis indicated that COL2 (*P* = 0.005), aggrecan (*P* = 0.001), SOX9 (*P* < 0.001), β1 integrin (*P* < 0.001), and p-mTOR (*P* = 0.004) expression increased significantly, but COL1 (*P* < 0.001) expression decreased significantly after 40 mW/cm^2^ intensity LIPUS stimulation of BMSCs treated with TGF-β1. After treatment with GRGDSP, COL2 (*P* = 0.015), aggrecan (*P* < 0.001), SOX9 (*P* = 0.008), COL1 (*P* = 0.005), β1 integrin (*P* = 0.001), and p-mTOR (*P* = 0.033) expression decreased significantly in the BMSCs. However, COL2 (*P* = 0.832), aggrecan (*P* = 0.916), SOX9 (*P* = 0.949), COL1 (*P* = 0.508), β1 integrin (*P* = 0.815), and p-mTOR (*P* = 0.954) expression did not change significantly when BMSCs were stimulated with 40 mW/cm^2^ intensity LIPUS stimulation after treatment with GRGDSP. After Rapamycin treatment, COL2 (*P* = 0.029), aggrecan (*P* = 0.004), SOX9 (*P* = 0.009), COL1 (*P* = 0.017), and p-mTOR (*P* < 0.001) expression decreased significantly, but the expression of β1 integrin (*P* = 0.884) did not change significantly in the BMSCs. Furthermore, 40 mW/cm^2^ intensity LIPUS following Rapamycin treatment of BMSCs caused β1 integrin (*P* = 0.013) expression to increase significantly but COL2 (*P* = 0.778), aggrecan (*P* = 0.424), SOX9 (*P* = 0.705), COL1 (*P* = 0.868), and p-mTOR (*P* = 0.788) expression did not change significantly (Fig. 5e–j).

## Discussion

The aim of this study was to determine whether LIPUS affects TGF-β1-mediated chondrogenic differentiation of BMSCs through the integrin-mTOR signaling pathway. We found that it significantly promoted chondrogenesis through the integrin-mTOR signaling pathway in BMSCs which had been treated with TGF-β1.

Many factors influence differentiation of BMSCs, and the regulation of growth factors plays an important role in the differentiation of BMSCs, in addition to interactions with the local environment and adjacent cells. Previous studies have confirmed that TGF-β can induce chondrogenic differentiation of BMSCs [19, 32, 33]. We found that culture medium containing TGF-β1 could induce chondrogenic differentiation of BMSCs, consistent with previous studies.

The effect of LIPUS on cells, such as chondrocytes, has been explored extensively [34]. Vaughan et al. found that LIPUS, applied for 20 minutes/day, stimulates the synthesis of sulphated glycosaminoglycans (sGAGs) in adult bovine articular chondrocytes [16]. Choi et al. demonstrated that increased viability and metabolism of human articular chondrocytes was induced by LIPUS treatment, suggesting that this could be a promising autologous source for cartilage tissue engineering [35]. Takeuchi et al. found that LIPUS promoted the proliferation of cultured chondrocytes and the production of type-IX collagen [36]. Ito et al. demonstrated that LIPUS inhibits mRNA expression of matrix metalloproteinase-13 (MMP-13) induced by interleukin-1 beta in chondrocytes [37]. Hasanova et al. suggested that a LIPUS stimulation regimen was shown to modulate the proliferative capacity, biosynthetic activity, and integrin mRNA expression of articular chondrocytes [38]. Nishikori et al. also found that LIPUS exposure promoted proliferation and chondroitin sulfate synthesis in cultured chondrocytes [39].

Although several studies have demonstrated that LIPUS stimulation of chondrocytes enhances cartilage matrix formation, few studies focused on the effects of LIPUS on the chondrogenic differentiation of BMSCs. Ebisawa et al. found that human MSCs treated with TGF-β differentiated into chondrocytes, whereas non-treated cells did not. Furthermore, when LIPUS was applied for 20 minutes every day to TGF-β-treated cells, chondrogenic differentiation was enhanced. Their results indicated that LIPUS significantly accelerated TGF-mediated chondrocyte differentiation as assessed by aggrecan deposition [19]. In our experiment, we found that the expression of COL2, SOX9, and aggrecan was significantly increased and COL1 expression significantly decreased in BMSCs that were treated with TGF-β1 when stimulated with LIPUS. As COL2, SOX9, and aggrecan are specific markers of chondrocytes and COL1 a specific marker of osteoblasts, we hypothesized that LIPUS could promote chondrogenic differentiation of BMSCs when treated with TGF-β1.

We further demonstrated that inhibition of integrins using GRGDSP suppressed COL2, aggrecan, SOX9, and COL1 expression and decreased the activity of mTOR in BMSCs which were treated with TGF-β1. Integrins are a family of cell surface stress receptors mediating cellular interactions with the ECM in addition to cell-cell interactions, and participates in the regulation of cell proliferation, differentiation and migration [40]. A previous study demonstrated that β1 integrin plays a key role in phenotypic maintenance and dedifferentiation of MSCs [41]. Thus, we speculated that integrins located upstream of mTOR, and inhibition of β1 integrin would suppress the chondrogenic differentiation of BMSCs.

Previous studies have demonstrated that the biological activities of BMSCs, such as differentiation can be activated by mechanical stress, which is mediated by integrins [21–23]. It has been found that LIPUS influences multilineage differentiation of BMSCs through various signaling pathways [17]. But the specific mechanism of LIPUS on the chondrogenic differentiation of BMSCs is not fully understood. Our previous study demonstrated that the effects of LIPUS on COL2 and aggrecan production in chondrocytes are mediated via the integrin signaling pathway [24]. In this study, we also found that the expression of β1 integrin in BMSCs treated with TGF-β1 increased significantly as did COL2, aggrecan, and SOX9 following LIPUS stimulation. These results suggest that the effects of LIPUS on chondrogenic differentiation of BMSCs might be via an integrin-mediated mechanotransduction pathway.

We found that the expression of p-mTOR increased significantly in BMSCs after stimulation using LIPUS. This suggests that when integrin is activated by LIPUS, downstream mTOR is activated in BMSCs. The activation of mTOR mainly increases the speed of the cell cycle and thus regulates proliferation. Thus, mTOR has an important role in regulating cell energy and metabolism, and so is the center of activity in the cell [42–46]. Therefore, we speculated that LIPUS may facilitate phosphorylation of mTOR in BMSCs in order to promote adaptation to moderate mechanical stress.

We found that, LIPUS stimulation had no significant effect on the expression of COL2, aggrecan, SOX9, COL1, and p-mTOR in BMSCs following addition of the integrin inhibitor GRGDSP, suggesting again that mTOR is downstream of the integrin pathway and that inhibition of integrin expression disrupts the effects of LIPUS on mTOR and chondrogenic differentiation of BMSCs. We also found that mTOR inhibition affected chondrogenic differentiation of BMSCs and the treatment effects of LIPUS. When the mTOR inhibitor, Rapamycin was used, the expression of COL2, aggrecan, and SOX9 decreased significantly in BMSCs, indicating that mTOR might be a positive factor in the chondrogenic differentiation of BMSCs. Our results also showed that following Rapamycin treatment, LIPUS stimulation had no significant effect on the expression of COL2, aggrecan, SOX9 and COL1 in BMSCs. Thus, we can deduce that mTOR is also a positive factor in the chondrogenic differentiation of BMSCs stimulated by LIPUS.

## Conclusions

In conclusion, LIPUS was found to affect TGF-β-mediated chondrogenic differentiation through the integrin-mTOR signaling pathway. Therefore, we deduce that the integrin-mTOR signaling pathway plays an important role in the promotional effects of LIPUS on chondrogenic differentiation in BMSCs. Our findings further uncover the mechanism of the effects of LIPUS on BMSCs and provide some basis for the development of OA treatment using BMSCs and LIPUS in the future.

## Abbreviations

ACI: Autologous chondrocyte implantation
ANOVA: Analysis of variance
BMSCs: Bone marrow mesenchymal stem cells
COL1: Collagen type-I
COL2: Collagen type-2
DMEM: Dulbecco’s modified Eagle’s medium
ECM: Extracellular matrix
FBS: Fetal bovine serum
FITC: Fluorescein isothiocyanate
GAPDH: Glyceraldehyde 3-phosphate dehydrogenase
GRGDSP: Glycine-arginine-glycine-aspartic acid-serine-proline
HRP: Horseradish peroxidase
IF: Immunofluorescence
IgG: immunoglobulin G
LIPUS: Low-intensity pulsed ultrasound
MACI: Autologous chondrocyte implantation
mTOR: Mechanistic target of rapamycin
OA: Osteoarthritis
PBS: Phosphate-buffered saline
SEM: Standard error of the mean
sGAGs: Sulphated glycosaminoglycans
SOX9: SRY-related high mobility group-box gene 9
TGF: Transforming growth factor
